# Treatment of hypercalcemia related to polymethylmethacrylate injection: report of three cases

**DOI:** 10.31744/einstein_journal/2025RC1377

**Published:** 2025-06-03

**Authors:** Lívia Reis de Miranda, Eduardo de Paiva Luciano, Eduarda Andrea Silva Rizzo, Marissol Fernandes Oliveira, Sheila Cavalca Cortelli, Walnei Fernandes Barbosa

**Affiliations:** 1 Universidade de Taubaté Taubaté SP Brazil Universidade de Taubaté, Taubaté, SP, Brazil.; 2 Universidade Federal de São Paulo São Paulo SP Brazil Universidade Federal de São Paulo, São Paulo, SP, Brazil.; 3 Universidade Estadual de Campinas Campinas SP Brazil Universidade Estadual de Campinas, Campinas, SP, Brazil.

**Keywords:** Polymethyl methacrylate, Hypercalcemia, Renal insufficiency, Cinacalcet, Aesthetic procedures

## Abstract

Polymethylmethacrylate is a microsphere-based plastic used in aesthetic procedures. However, it can cause granuloma formation and severe hypercalcemia, often accompanied by renal impairment, which is a significant concern. Despite increasing reports, there is no standardized treatment approach for polymethylmethacrylate-induced hypercalcemia, and its incidence remains underreported. In this study, we present three cases of polymethylmethacrylate users who developed severe hypercalcemia that was resistant to conventional treatments, resulting in deteriorating renal function. These patients achieved symptom control with cinacalcet, a medication primarily used to treat secondary hyperparathyroidism, severe primary hyperparathyroidism, and parathyroid cancer.

## INTRODUCTION

In 2022, Brazil experienced a substantial 390% increase in the demand for non-surgical aesthetic procedures.^(1)^

Polymethylmethacrylate (PMMA) is one of the most widely used permanent fillers for these procedures because of its ease of application and long-lasting effects, despite its restricted approval by the National Health Surveillance Agency (ANVISA - *Agência Nacional de Vigilância Sanitária*) exclusively for the correction of lipodystrophy and volumetric facial and body defects.^(2)^ Unlike reabsorbable substances such as collagen and hyaluronic acid, PMMA provides a permanent correction, enhancing its aesthetic appeal. However, its application carries risks of complications, including inflammation, nodules, necrosis, and in 0.2–1% of cases, granuloma formation, often associated with excessive volume or incorrect application.^(3)^

The first reported case of hypercalcemia associated with PMMA administration dates back to 2014. Macrophages within PMMA-induced granulomas may produce extra-renal CYP27B1 (alpha 1 hydroxylase), leading to increased calcitriol production and subsequent hypercalcemia.^(4)^ Subsequent case reports have also highlighted the severity and often irreversible kidney damage associated with PMMA-induced hypercalcemia; however, a definitive and effective treatment remains elusive.

Cinacalcet, a calcimimetic agent, is primarily used to treat secondary hyperparathyroidism in patients with chronic kidney disease, as well as severe primary hyperparathyroidism and parathyroid cancer. It lowers parathyroid hormone (PTH) levels by increasing the sensitivity of the parathyroid calcium-sensing receptor (CaSR) to extracellular calcium.^(5)^ As its primary mechanism of action in the treatment of parathyroid cancer, cinacalcet may interact with CaSR in the bone and/or kidneys. No clinical trials have yet confirmed its effectiveness in treating hypercalcemia secondary to granuloma activation following PMMA injection; however, there are still no clinical trials to support the hypothesis of cinacalcet action.^(6)^

Given the limited treatment options for PMMA-induced hypercalcemia, this study aimed to explore the potential role of cinacalcet in its management. This study aimed to determine whether cinacalcet is a viable therapeutic alternative to conventional interventions, thereby improving patient outcomes.

## CASE REPORT

### Case 1

A 55-year-old female presented to our medical care department in September 2020 with nausea and deteriorating kidney function following antibiotic therapy for *Helicobacter pylori* infection. Laboratory findings on admission revealed hypercalcemia (serum calcium: 14mg/dL; ionic calcium: 1.81mmol/L), impaired renal function (creatinine: 1.57mg/dL), anemia, and an initial suspicion of multiple myeloma, although no monoclonal components were detected. Symptoms were managed with hydration, antiemetics, and zoledronic acid, followed by outpatient monitoring. At her initial outpatient visit, she presented with persistent nausea, uncontrolled vomiting, and recent weight loss, while denying any other symptoms. Laboratory tests revealed anemia (hemoglobin, 8.5g/dL) with a normal MCV and negative tumor markers. PTH and 1,25 dihydroxyvitamin D levels were within normal limits (Table 1). Beta-2 microglobulin was slightly elevated at 3.6mg/L (VR 0.60 – 2.45mg/l).

**Table 1:** Initial laboratory presentation

Patients	Calcium (mg/dL)	Albumin (g/dL)	Creatinine (mg/dL)	ionized calcium (mmol/L)	Phosphorus (mg/dL)	25D (ng/mL)	1.25D (pg/mL)	24-hour urinary calcium (mg/24h)	Angiotensin-converting enzyme (13.3 a 64 mg/ml)
Case 1	14.0	4.1	1.57	1.81	2.8	59.8	67.5	414	54.7
Case 2	11.0	4.2	1.35	1.58	NA	45.7	95.3	202	49.5
Case 3	12.2	3.8	1.78	1.65	NA	NA	102.5	186	66.7

NA: not available.

**Figure 1:**
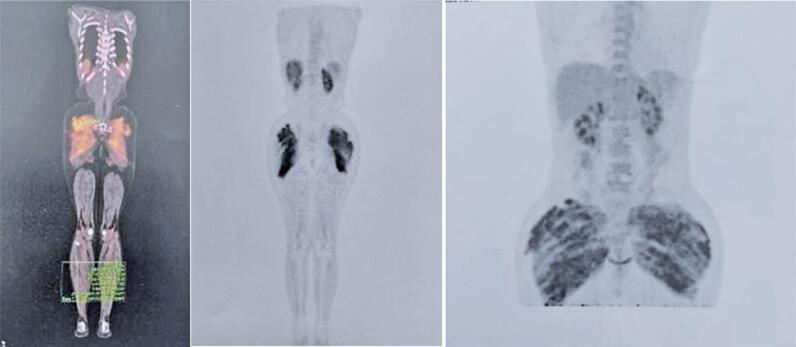
PET CT images in case 1

Serum and urine electrophoresis and immunofixation revealed no monoclonal peaks, and the light-free kappa/lambda ratio was normal. Mild proteinuria was detected at 280mg/dL (VR <150mg/dl). The patient had a history of anorexia and cosmetic surgeries. She also reported using hormone replacement therapy and supplements containing calcium, vitamin D, vitamin K and magnesium. Following the consultation and based on the clinical findings, provisional diagnoses of exogenous calcium poisoning, non-secreting myeloma, or paraneoplastic syndrome were made. Additional investigations were initiated, and zoledronic acid therapy was continued because of the persistently elevated calcium levels. Two months later, the patient reported symptomatic improvement and a benign physical examination. Laboratory results indicated partial improvement in anemia (hemoglobin, 10.9g/dL). Renal function tests showed slight impairment, with a creatinine clearance of 60mL/min (reference value >85ml/min), urea of 37mg/dL (reference value <40 mg/dl), and creatinine of 0.9 mg/dl (reference value <1.2mg/dl). Hypercalcemia improved, with a serum calcium level of 10.2mg/dl (reference range 8.8-10.4mg/dL) and ionized calcium of 1.27mmol/L (reference range 1.17–1.30mmol /L). PTHrp level was normal. Urinalysis revealed hypercalciuria, with a urinary calcium level of 414mg/day (reference range, 55–220mg/day). Bone X-rays revealed no abnormalities. During this consultation, the patient disclosed prior PMMA injections for aesthetic purposes, administered approximately 8 months before symptom onset. Given this history, along with the test results excluding other causes of hypercalcemia and the existing literature linking hypercalcemia to PMMA application, a PMMA-associated foreign body granuloma was suspected. A PET CT scan revealed active granulomas in the buttocks (Figure 1) and face. The patient received zoledronic acid monthly for 11 months, occasionally with furosemide, and initially achieved partial control. However, the treatment response diminished despite the addition of prednisone in the last 2 months of treatment, leading to deteriorating renal function. Denosumab was subsequently initiated; however, effective control was not achieved after 4 months of therapy.

**Figure 2:**
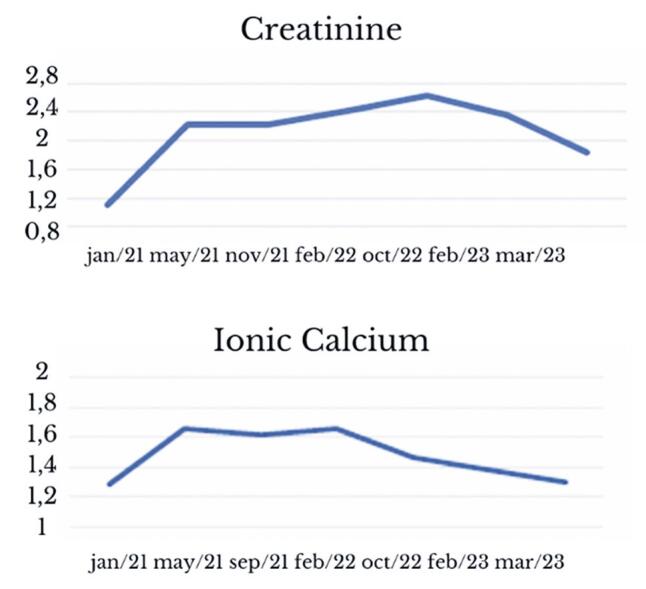
Laboratory evolution in case 1

The nephrology team evaluated alternative treatment options. Despite the limited understanding of the direct role of cinacalcet in bone metabolism, the presence of CaSR in bone and kidney tissues suggests that it plays a potential role in calcium regulation. Consequently, cinacalcet was recommended as an alternative therapy for patients with SHPT. Treatment was initiated with half a tablet every other day, increasing to daily use once it was well-tolerated by the patient. Following initiation, there was a decrease in serum calcium levels over 3 months from 11.6mg/dL (prior to treatment) to 10.3mg/dL, with sustained improvement even after 6 months, stabilizing renal function and alleviating symptoms throughout the follow-up period (Figure 2).

Currently, the patient has been using cinacalcet for 2 years, with ionized calcium =1.18; creatinine = 1.72mg/dL; and an estimated glomerular filtration rate (eGFR) of 38mL/min (Table 2).

**Table 2:** Laboratory evolution of patients after starting cinacalcet

iCa/Creat (mmol/l)/(mg/dL)	3 months	6 months	1 year
Case 1	1.75/2.5	1.45/2.0	1.35/2.3
Case 2	1.58/2.3	1.35/1.8	1.30/1.4
Case 3	1.78/3.4	1.44/1.8	1.35/1.1

iCa: Ionic calcium; creat: Creatinine.

### Case 2

**Figure 3:**
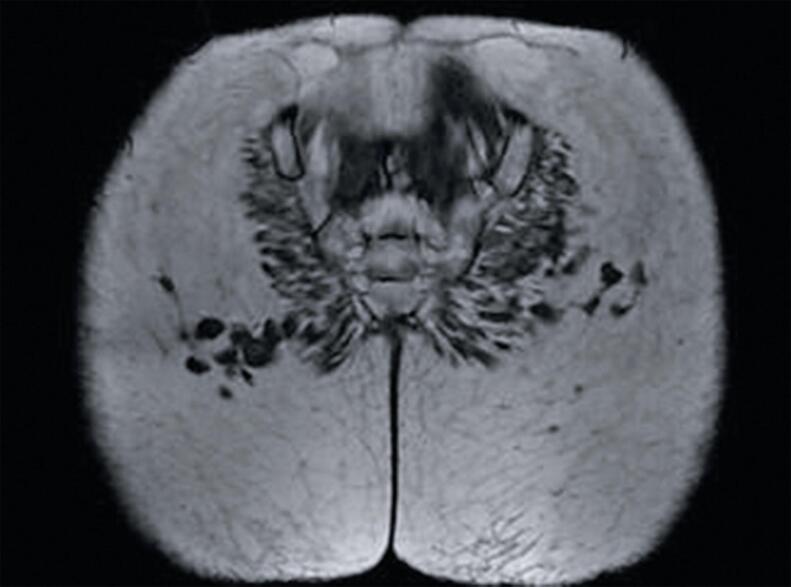
Case 2 - MRI image of gluteal granulomas

The second patient was a 45-year-old female who had been monitored for Kikuchi-Fujimoto syndrome for 6 years and was referred by her rheumatologist for medical evaluation. She reported receiving PMMA injections in the buttocks 8 years prior to consultation. At the time of evaluation, she was in remission but presented with hypercalcemia (ionic calcium: 1.58), polyuria, polydipsia, and renal function alterations. Magnetic resonance imaging (MRI) showed granulomas in the buttocks (Figure 3).

Initial treatment with bisphosphonates followed by denosumab failed to achieve an effective response. Based on the clinical and laboratory findings, a diagnosis of hypercalcemia secondary to ectopic calcitriol production was established. Cinacalcet therapy was initiated at a dose of 15mg/day, resulting in sustained normalization of ionic calcium levels, improved renal function, and symptom relief. Kidney function of the patient is currently normal, with ionic calcium levels of 1.20mEq/L while receiving cinacalcet at a maintenance dose of 30mg every 3 days.

### Case 3

The third case involved a 40-year-old male with a history of PMMA injection into the buttocks 6 years ago. He sought medical attention because of symptoms of polyuria, and initial admission tests revealed an estimated glomerular filtration rate (eGFR) of 34mL/min and hypercalcemia with a serum calcium level of 1.78mmol/L (Table 1).

Despite previous treatment with zoledronic acid, furosemide, corticosteroids, and denosumab, no clinical or laboratory improvement was observed. Consequently, cinacalcet treatment was initiated at a dose of 7.5mg daily, which was subsequently increased to 15mg daily. This regimen resulted in the normalization of serum calcium levels and stabilization of the renal function (Table 2).

## DISCUSSION

Polymethylmethacrylate is approved exclusively for the correction of lipodystrophy; however, it has been widely used for aesthetics by non-medical professionals and inadequately trained doctors because of its affordability and lack of strict regulatory oversight.^(2,7)^ Its irregular surface prevents phagocytosis, often leading to the formation of granulomas. Studies have shown that this complication occurs in approximately 0.1% to 1% of patients, typically developing 6–24 months post-implantation at the site of injection, although cases have been reported up to 10 years later.^(7)^

One of the contributing factors to PMMA-associated complications is the presence of impurities in its formulation, which was used in Brazil before 2006. According to the Brazilian Society of Plastic Surgery (SBCP, 2022), between mid-2015 and 2016, approximately 17, 000 patients sought corrective procedures from plastic surgeons owing to PMMA sequelae. There have been a few documented cases of hypercalcemia associated with PMMA, with the earliest reports dating back to 2014.^(8)^

The diagnostic criteria for PMMA-associated complications remain unclear and are often based on exclusion criteria. Imaging techniques, particularly those capable of detecting inflammation, are promising diagnostic techniques. Chronic granulomas increase the conversion of calcifediol to calcitriol, leading to enhanced intestinal calcium absorption, bone resorption, hypercalcemia, hypercalciuria, and suppression of PTH levels. As this conversion is extra-renal in origin, these changes occur despite normal PTH, sex steroid, and phosphate levels.^(8,9)^Imaging tests, such as MRI, can identify granulomas; however, granulomas are present even in patients without hypercalcemia. For instance, a reported case described a patient presenting with hypercalcemia symptoms approximately eight months post-PMMA application, with metabolic and etiological factors initially ruled out. PET CT imaging confirmed a granulomatous activity at the PMMA injection sites, suggesting its potential utility in complex diagnostic cases.

A study by Tachamo et al evaluated various treatment modalities for PMMA-induced hypercalcemia, including hydration, corticosteroids, bisphosphonates, and denosumab, each showing variable responses, with frequent hypercalcemia recurrence observed in approximately 82.35% of cases. Reports have also documented attempts with prednisone and denosumab alone; however, hypercalcemia persisted, and renal function deteriorated, necessitating dialysis.^(10,11)^

Currently, no established therapy consistently achieves a sustained remission. Standard management involves saline hydration, intravenous bisphosphonates, and glucocorticoids. Glucocorticoids counteract the effects of 1,25(OH)2D and suppress the inflammatory mediators that activate CYP27B1; however, they often yield only transient responses and may cause side effects.^(11,12)^

Cinacalcet, a calcimimetic, interacts allosterically with CaSR on parathyroid cells, reducing PTH secretion and lowering the serum calcium levels. As mentioned previously, CaSR is also present in bone and kidney tissues, and although there is still a relatively limited understanding of its direct role in bone metabolism, it plays an important role in renal calcium handling.^(6)^

No clinical trials have investigated the use of cinacalcet for treating hypercalcemia associated with malignancies. The postulated primary mechanism of action in the treatment of parathyroid cancer suggests a possible interaction between cinacalcet and CaSR in the bone and/or kidneys. Oral cinacalcet may be an efficacious therapy for malignancy-related hypercalcemia to elevate 1,25-dihydroxyvitamin D. Furthermore, it is hypothesized that this effect is primarily mediated by the interaction of cinacalcet with CaSR in the intestine, with lesser effects on the bones and kidneys. Finally, the role of 1,25-dihydroxyvitamin D in hypercalcemic malignancies may be underappreciated in solid tumors. No clinical trials have supported the hypothesis of cinacalcet treatment of hypercalcemia secondary to granuloma activation after PMMA injection. Based on this rationale, we hypothesized that cinacalcet acts on the calcium-sensitive receptors of 1,25-dihydroxyvitamin D-producing granulomas.^(6,13)^

Regarding extra-renal synthesis and the non-classical action of 1,25-(OH)2D, studies have shown that monocytes, dendritic cells (DCs), macrophages, B-cells, and T-cells possess 25(OH)D-1-α hydroxylase activity to locally transform 25(OH)D into 1,25-(OH). Similar to its classical action, the immunomodulatory effects of vitamin D (non-classical action) are mediated by the vitamin D receptor in immunomodulatory cells. This immunomodulatory function plays a key role in the pathogenesis of granulomatous disorders, such as sarcoidosis and tuberculosis.^(14,15)^

Unlike renal (CYP27B1) activity, extra-renal 25(OH)D-1-α hydroxylase CYP27B1 activity is highly substrate-dependent, and its production is significantly affected by the prevailing serum concentration of 25(OH)D. Therefore, the local production of 1,25-(OH)2D is deficient when the serum concentration of 25(OH)D is low (below 25 ng/mL).^(15)^

In the presented cases, standard therapies such as zoledronic acid and prednisone were ineffective in achieving sustained control. In the absence of established guidelines, cinacalcet treatment was initiated, resulting in effective control of hypercalcemia and improvement in renal function. This approach appears to prevent the need for renal replacement therapy, which is a common outcome in such patients. Nausea was the primary side effect of cinacalcet, which was managed by adjusting the dosage and antiemetic drugs. Combination therapy with other modalities may be considered for refractory cases or those with intolerable side effects and requires further study.

## CONCLUSION

This study demonstrates that polymethylmethacrylate -induced hypercalcemia remains poorly documented and is possibly underdiagnosed. Currently, treatment options are not well established, and reported cases often raise concerns about renal outcomes. As the use of polymethylmethacrylate continues to rise, the urgent need for effective treatment becomes increasingly evident. These findings provide critical insights into the challenges associated with polymethylmethacrylate complications, highlighting the potential role of calcimimetic agents in mitigating kidney injuries. In this report, the use of a calcimimetic agent mitigates kidney injury. Long-term follow-up and further studies are essential to investigate the efficacy and safety of these medications and establish reliable treatment strategies for severe complications associated with polymethylmethacrylate. Future research should investigate the safety and efficacy of calcimimetic agents through large-scale clinical trials to validate and extend these findings. By addressing these challenges, this study establishes a foundation for improved clinical management of polymethylmethacrylate-induced hypercalcemia, potentially enhancing patient outcomes and guiding future treatment strategies.
